# High-Throughput, Amplicon-Based Sequencing of the CREBBP Gene as a Tool to Develop a Universal Platform-Independent Assay

**DOI:** 10.1371/journal.pone.0129195

**Published:** 2015-06-09

**Authors:** Marc W. Fuellgrabe, Dietrich Herrmann, Henrik Knecht, Sven Kuenzel, Michael Kneba, Christiane Pott, Monika Brüggemann

**Affiliations:** 1 2nd Medical Department, University Hospital Schleswig-Holstein, Kiel, Germany; 2 Max-Planck Institute for Evolutionary Biology, Ploen, Germany; Cairo University, EGYPT

## Abstract

High-throughput sequencing technologies are widely used to analyse genomic variants or rare mutational events in different fields of genomic research, with a fast development of new or adapted platforms and technologies, enabling amplicon-based analysis of single target genes or even whole genome sequencing within a short period of time. Each sequencing platform is characterized by well-defined types of errors, resulting from different steps in the sequencing workflow. Here we describe a universal method to prepare amplicon libraries that can be used for sequencing on different high-throughput sequencing platforms. We have sequenced distinct exons of the CREB binding protein (CREBBP) gene and analysed the output resulting from three major deep-sequencing platforms. platform-specific errors were adjusted according to the result of sequence analysis from the remaining platforms. Additionally, bioinformatic methods are described to determine platform dependent errors. Summarizing the results we present a platform-independent cost-efficient and timesaving method that can be used as an alternative to commercially available sample-preparation kits.

## Introduction

In the last years, high-throughput sequencing has become a cost-efficient method to generate multiple sequencing data from different genomic fragments, thereby opening new opportunities in the field of genomic research. The high-throughput sequencing (HTS)-platforms had their breakthrough in 2005 with the 454 pyrosequencing-based technology [1], followed by Illumina array-based sequencing-by-synthesis approach with reversible-terminator chemistry in 2007 [2,3]. Life Technologies launched the Ion Torrent semiconductor chip-based Personal Genome Machine (PGM) in 2011 [4]. An informative overview about technical features and sequencing chemistry of these commercially available high-throughput sequencing platforms is reviewed by Metzker [5].

All these technologies enable a fast and cost-effective alternative to Sanger sequencing [6], which has been the gold standard over the last decades. Moreover high-throughput data are produced within a short period of time, while the sequencing costs have been markedly reduced. Recent studies validate the performance of these platforms by comparing whole-genome sequencing data, demonstrating platform specific limitations [7–9]. Different types of errors may appear during the whole amplification and sequencing process, with platform-specific error rates and dominant error types (e.g. substitution, insertion, deletion), corresponding to the sequencing chemistry used [10].

We focused on the three major platforms, which are currently available on the market:. The 454 GS Junior/FLX instruments provide the longest reads (up to 1000 bp for GS FLX+) and high-quality sequences, but they are very expensive due to their emulsion PCR and pyrosequencing chemistry. In contrast the IonTorrent PGM has the highest throughput but the lowest base accuracy. Both platforms show specific problems in homopolymeric regions, where a long stretch of the identical nucleotides generates predominantly Indel errors [11]. In contrast, Illumina MiSeq produces the shortest reads (2 x 250 bp), but by using four labeled-reversible terminators, and due to a long run-time (27 h) the most accurate data are generated. Furthermore, PGM and Illumina technologies have been reported to be error prone to distinct sequence motifs [9].

Here we report a strategy to overcome the individual disadvantages of single high-throughput sequencing platforms, and to use the full potential of these technologies by obtaining high-accuracy data of the analysed amplicon sequences. We established a universal amplicon-sequencing strategy for amplification of targets in a two-step PCR approach, with the first PCR being gene-specific and platform-independent whereas the second-step PCR links the amplicons to the platform-specific adapters. Results of comparative analysis, deriving from this sequencing approach, were used to determine quality and error rates of the platforms tested.

## Materials and Methods

### Amplicon library preparation

Mutational analysis of five distinct exonic regions of the CREBBP gene (ENSEMBL Gene ID: ENSG00000005339; Chr 16:3775055–3930727) was performed from genomic DNA samples of unsorted mononuclear cells from ten patient samples by PCR amplification. Samples were obtained during reference diagnostic within the German Multicenter ALL Study Group (GMALL) trial 07/2003 (Clinicaltrials.gov identifier:00198991). Patients agreed with the use of left-over material for research purposes. Approval for this study was obtained from the ethical board of the Medical Department at the Johann Wolfgang Goethe-University, Frankfurt am Main, Germany. Our study complied with the principles set forth in the Declaration of Helsinki.

**Fig 1:**
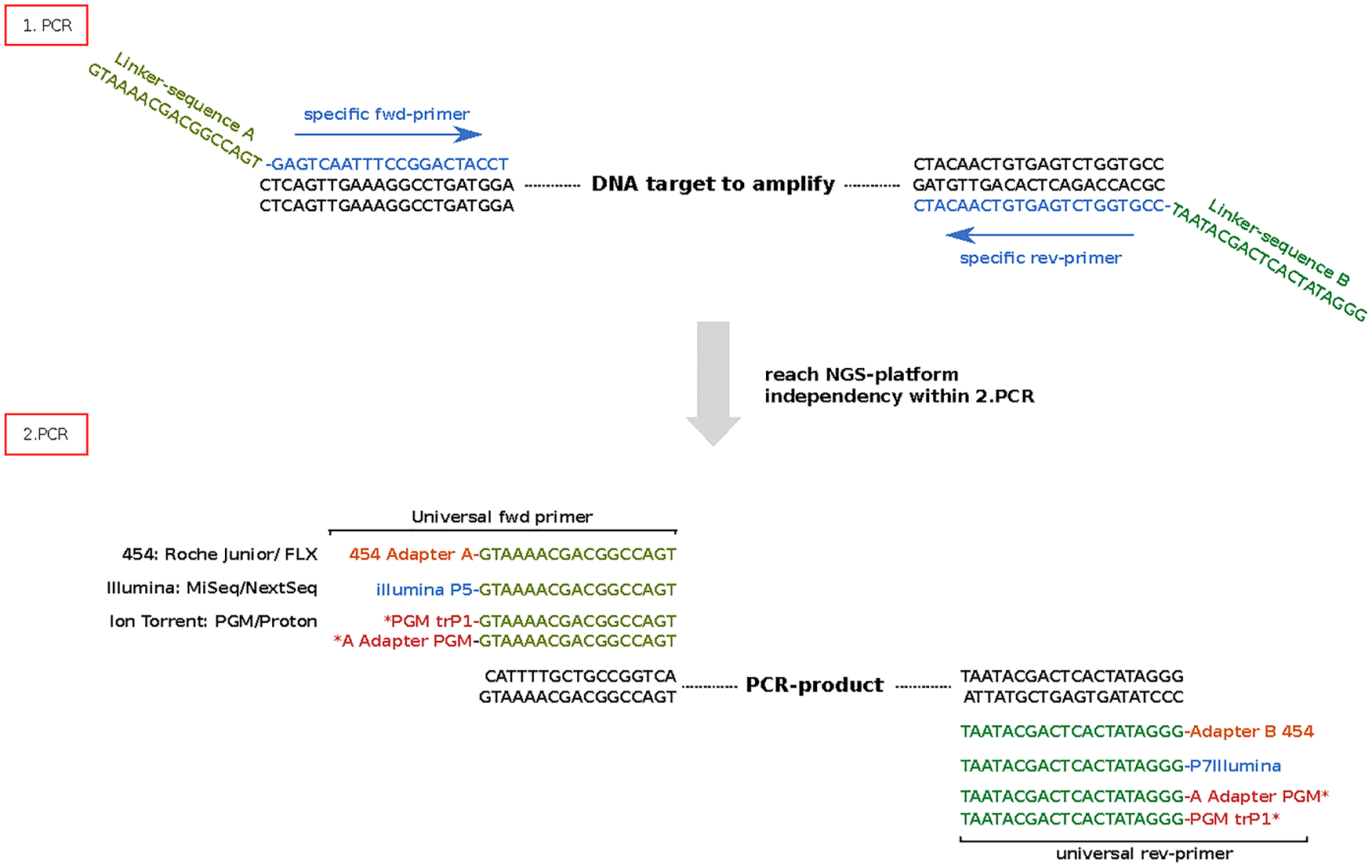
Universal two-step PCR approach. First-round PCR is performed by using gene-specific primers tailed with a universal linker sequence (light and dark green). The universal tailed amplicons can be used for second-round PCR, where deep sequencing platform-specific adapters can be introduced for either PGM (dark red), Illumina (blue), or 454 (orange). *To prepare PGM amplicon libraries containing trP1 and A adapter sequences on both ends of the library the second-round PCR has to be separated in two reactions with different primer combinations, respectively. All platform-specific amplicon libraries can be prepared with unique barcodes to sequence multiple samples.

The amplification workflow is shown in Fig 1. Primers were designed to amplify 325–370 bp regions, whereas forward and reverse primers were tailed with univ- and T7-linker sequences, respectively. PCR was performed using FastStart High Fidelity PCR System (Roche) with 2 U Fast Start High-Fidelity Polymerase, 1.8 mM MgCl_2_, 0.2 mM each dNTP, and 0.4 μM of each primer. PCR thermocycler profile used was 95°C for 2 minutes, 35 cycles of 95°C for 30 seconds, 55°C for 30 seconds and 72°C for 30 seconds, followed by a final extension at 72°C for 4 minutes.

Different platform-specific primers were used for the second-round amplification (Fig 1). All samples were barcoded with either MID 1–10 (454 GS FLX), Barcode 1–10 (Ion Torrent PGM), or indexes D701-704 x D501-504 (Illumina MiSeq), respectively. PCR reactions were performed using 1 μl of a 1/10 diluted first-round PCR product as template and Fast Start High Fidelity PCR System (Roche) with 2 U Fast Start High-Fidelity Polymerase, 1.8 mM MgCl_2_, 0.2 mM each dNTP, and 0.2 μM of each primer. PCR thermocycler profile used was 95°C for 2 minutes, 20 cycles of 95°C for 30 seconds, 63°C for 30 seconds and 72°C for 30 seconds, followed by a final extension at 72°C for 5 minutes.

To prepare an amplicon library for bidirectional sequencing with the PGM, four fusion primers were required, thereby introducing the A adapter region to the proximal end and the trP1 adapter region to the distal end of the amplicon library. By using the second primer pair, adapters are introduced the other way around. 454 and Illumina amplicon library preparation can be performed in one reaction, thereby linking the universal adapter sequences and barcodes on both sites. Multiplexing is performed by fusing the barcode tag to the second-round PCR primer [12]. These barcodes are commercially available for all platforms—10 bp ‘multiplex identifiers’ (MID) are used by 454, as well as Ion Xpress barcodes by IonTorrent, and 8 bp indexes by Illumina that uses a ‘dual-index’ strategy.

All amplicons were purified twice by AMPure XP beads (Beckman Coulter) with a bead to DNA ratio of 0.75:1 to remove small DNA fragments. Quality control of the amplicon library was assessed by Agilent 2100 Bioanalyzer analysis. Quantitation of single amplicons was performed with Quant-it PicoGreen dsDNA Assay Kit (Invitrogen) on Lightcycler 480 II system (Roche), afterwards all amplicons were pre-diluted according to platform-specific concentrations, equimolarly pooled and applied to platform-specific sequencing workflow.

### Sequencing

#### Illumina MiSeq

The amplicon library was prepared for sequencing according to the manufacturer’s instructions (MiSeq Sequencing protocol). In brief, the amplicon pool was pre-diluted to 2 nM and denatured. 10% of denatured PhiX control (Illumina, # FC-110-3001) was spiked in 6 pM amplicon pool and loaded to the sample reservoir of the MiSeq Reagent Cartridge. We used the 500-cycle v2 kit for amplicon sequencing. The run was set by using the MiSeq Control Software (MiSeq Reporter 2.2.29) according to the *MiSeq System User Guide Part* (# 15027617). A sample sheet (csv-file) for FASTQ format output was designed with the Illumina Experiment Manager 1.5. The run was performed for 2 x 250 bp using a paired-end approach, with automated cluster-generation, Sequencing-by-synthesis (SBS) included reversible terminator sequencing of the indexed samples, and producing demultiplexed FASTQ files.

#### Ion Torrent PGM

Ion Torrent sequencing was performed according to the Ion Torrent standard workflow at Life Technologies lab in Darmstadt, Germany. Using the Agilent 2100 Bioanalyzer quantitation results, all barcoded-amplicons were diluted to 26 pM and equimolarly pooled. 25 μl of amplicon library was used for downstream application using the Ion PGM Template OT2 400 Kit (MAN 00007218) for use with the Ion OneTouch 2 System. This procedure resulted in the generation of template-positive Ion PGM Template OT2 400 Ion Sphere Particles. After enrichment of the particles, a sequencing run was performed with the Ion 318 chip V2 based on the Ion PGM Sequencing 400 Kit User Guide (MAN 00007242). FASTQ-files format was generated with Torrent suite software 3.6.

#### 454 GS FLX

454 GS FLX sequencing was performed at the Max Planck Institute for Evolutionary Biology in Ploen, Germany. After quantitation of single amplicons with Quant-it PicoGreen dsDNA Assay Kit (Invitrogen) on the Lightcycler 480 II system (Roche), single amplicons were diluted to 1 x 10^9 molecules and equimolarly pooled. A working dilution of 1x 10^7 molecules was prepared for downstream application. emPCR was performed with the GS FLX Titanium SV emPCR Kit (Lib-A) including emulsification, amplification, bead recovery, and DNA library bead enrichment, followed by the sequencing reaction according to the standard protocols. SFF files containing read base calls and per base quality scores were used for further analysis.

#### Data availability

Sequencing reads generated during this project have been deposited at the NCBI Sequence Read Archive (SRA) under the accession number SRP056569.

### Bioinformatics

#### Pre-Processing of 454 reads

All sequences generated by the 454 GS FLX platform were pre-processed by using SFF-Tools—sfffile command (v. 2.9) to separate reads by different MID and to assign amplicon reads to individual samples.

#### Quality Assessment

We used FastQC (http://www.bioinformatics.bbsrc.ac.uk/projects/fastqc) to assess HTS data quality. This tool is compatible with all main sequencing platforms, and quickly generates graphs for analysing ‘per base quality scores’ and ‘read length distribution’. Information of FastQC results were used for filtering and trimming sequencing reads with PRINSEQ tools [13]. As base-calling algorithms we used Phred quality score to estimate error probability for each base calling [14]. All bases with a Phred quality score < 20 were replaced by N, whereas all reads with a mean Phred quality score < 25 or reads containing more than 6 N bases were removed from downstream analysis.

#### Read Alignment

Reads passing PRINSEQ filter criteria were aligned to CREBBP gene information by using sequence alignment software BWA (bwa-0.6.2) [15,16]. Single reads resulting from 454 and IonTorrent platform were analysed with command bwa-sw; Illumina’s paired-end reads were analysed with bwa—aln and—sampe commands, resulting both in BAM file format.

#### Error rate identification

SAMtools (samtools-0.1.19) summarizes several tools for manipulating SAM and BAM files [17]. All reads were sorted and indexed with the commands—sort and—index. With the command—mpileup—uBD BAM files were further processed. Using toolbox BCFtools SNPs that is part of BWA tools, pre-processed BAM files were used to generate bcf-files by using command—view—bvcg. We used the analysis pipeline from Jünemann *et al*. to calculate the substitution and Indel error rates [18].

#### Substitution Matrix

Custom Perl scripts were used to calculate a substitution matrix from bcf-files generated by SAMTools and bcftools commands in the error rate identification pipeline. Here the total number of individual bases was divided by the number of substituted bases to identify base-specific errors.

## Results

### HTS universal two-step approach

We performed deep-sequencing on three different platforms and analysed five distinct exons of the CREBBP gene to investigate error rates. The CREBBP target gene is known to harbor mutational hotspot regions predominantly accomplished with hematological diseases [19]. The preparation of the amplicon libraries was performed in a two-step PCR procedure, where universal linker sequences were introduced by using 5’ tailed-primers which enables the connection of the platform-specific sequences in the second-round PCR (Fig 1). This technique enables preparation of libraries for all three platforms starting from the same DNA sample, thereby reducing the total amount of required input DNA for analysis. All amplicon-based libraries were tagged with a platform-specific set of barcodes (MID / Index / Barcode) to enable discrimination of different samples after the sequencing run.

### Performance of HTS platforms

In the first analysis we focused on base accuracy, read length distribution, number of reads and total number of generated bases for each investigated platform. As expected, large differences were detected between the three platforms. The 454 GS FLX system yielded the lowest number of reads (942,336) and sequenced bases (318 Mb), whereas the Illumina MiSeq had the highest throughput with 14,871,772 reads and 3.7 Gb produced. The IonTorrent PGM output yield was intermediate of the other platforms with 5,106,749 reads and 1.3 Gb (Table 1).

**Table 1:** Platform comparison—run details.

Platform	Number of reads	Total bases	Modal read length in bases	Mean read length in bases (sd)
**454 GS FLX**	942,336	318,141,622	325	337 (28)
**Ion Torrent PGM (318)**	5,106,749	1,268,444,165	325	248 (111)
**Illumina MiSeq**	14,871,772	3,718,004,124	251	250 (6)

GS FLX produced the longest reads with a mean read length of 337 bp, whereas both PGM and MiSeq system resulted in a mean read length of 250 bp each. Interestingly, the PGM platform delivered a high number of short reads, whereas the modal read length was 325 bp. These results confirmed that Illumina MiSeq has the highest throughput of all platforms tested.

**Fig 2:**
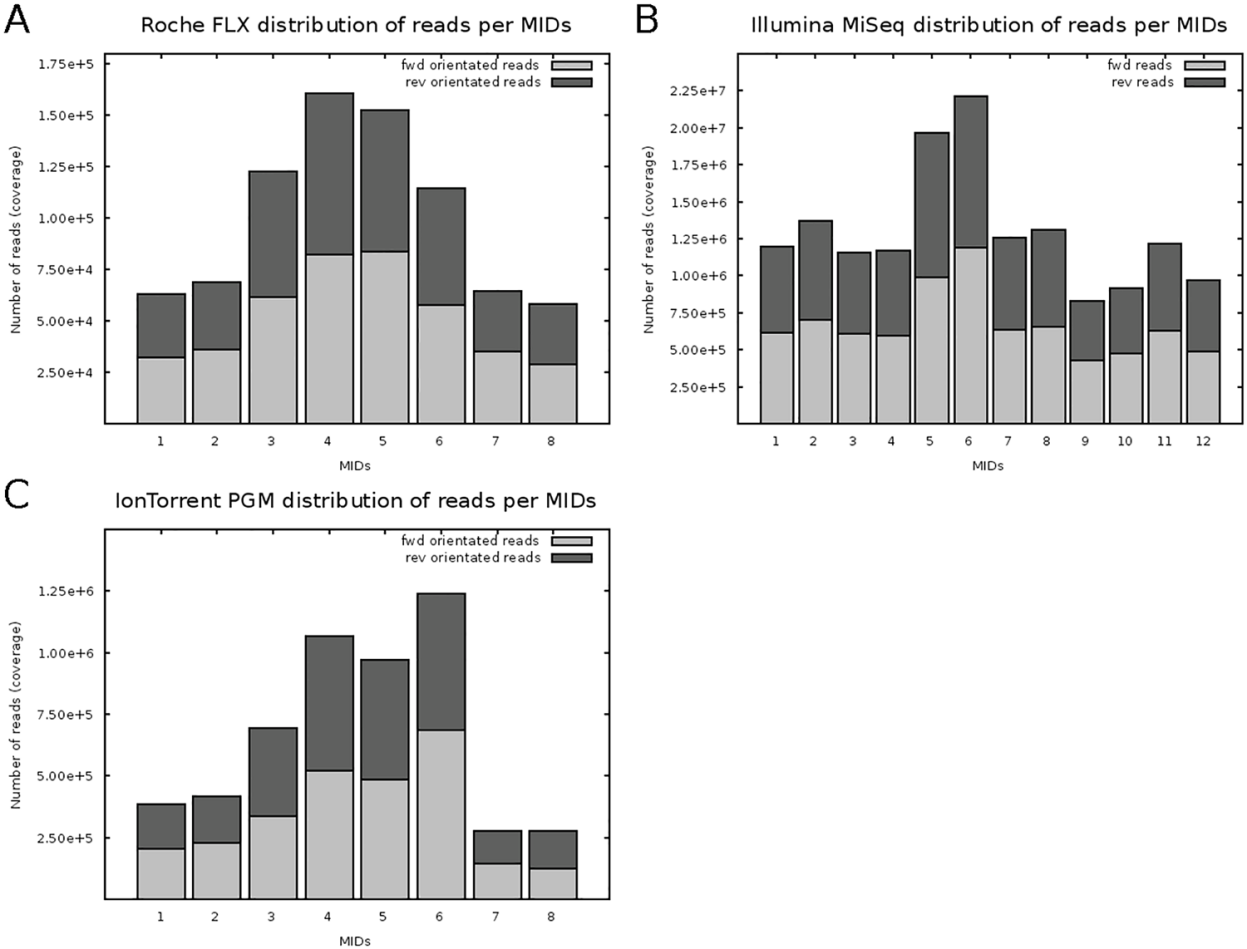
Platform comparison—read distribution. Total number of reads (coverage) for each single barcoded amplicon library generated by 454 (A), Illumina (B), and IonTorrent (C) platform are shown in both forward (light grey) and reverse (dark grey) read orientation.

All reads were separated by amplicon / sample-specific barcodes using specific algorithms (e.g. sff-tools) for each sequencing platform. The total number of reads for each sample and platform was plotted in Fig 2, separated by forward and reverse read orientation and in case of Illumina paired-end forward and reverse reads (light or dark grey bars). For all three platforms there was a homogenous distribution of forward- and reverse-orientated reads. Moreover, there slight difference in distribution of single amplicons were seen within individual platforms reaching 50,000–160,000 reads / amplicon for GS FLX (Fig 2A), 800,000–2,200,000 for MiSeq (Fig 2B), and 200,000–1,200,000 for PGM (Fig 2C), respectively.

As expected, there was no significant difference between the three platforms concerning preferential amplification of single amplicons, demonstrating reproducible and platform-independent sequencing reactions. The yield of forward- and reverse-orientated reads was balanced for all sequencing runs.

**Fig 3:**
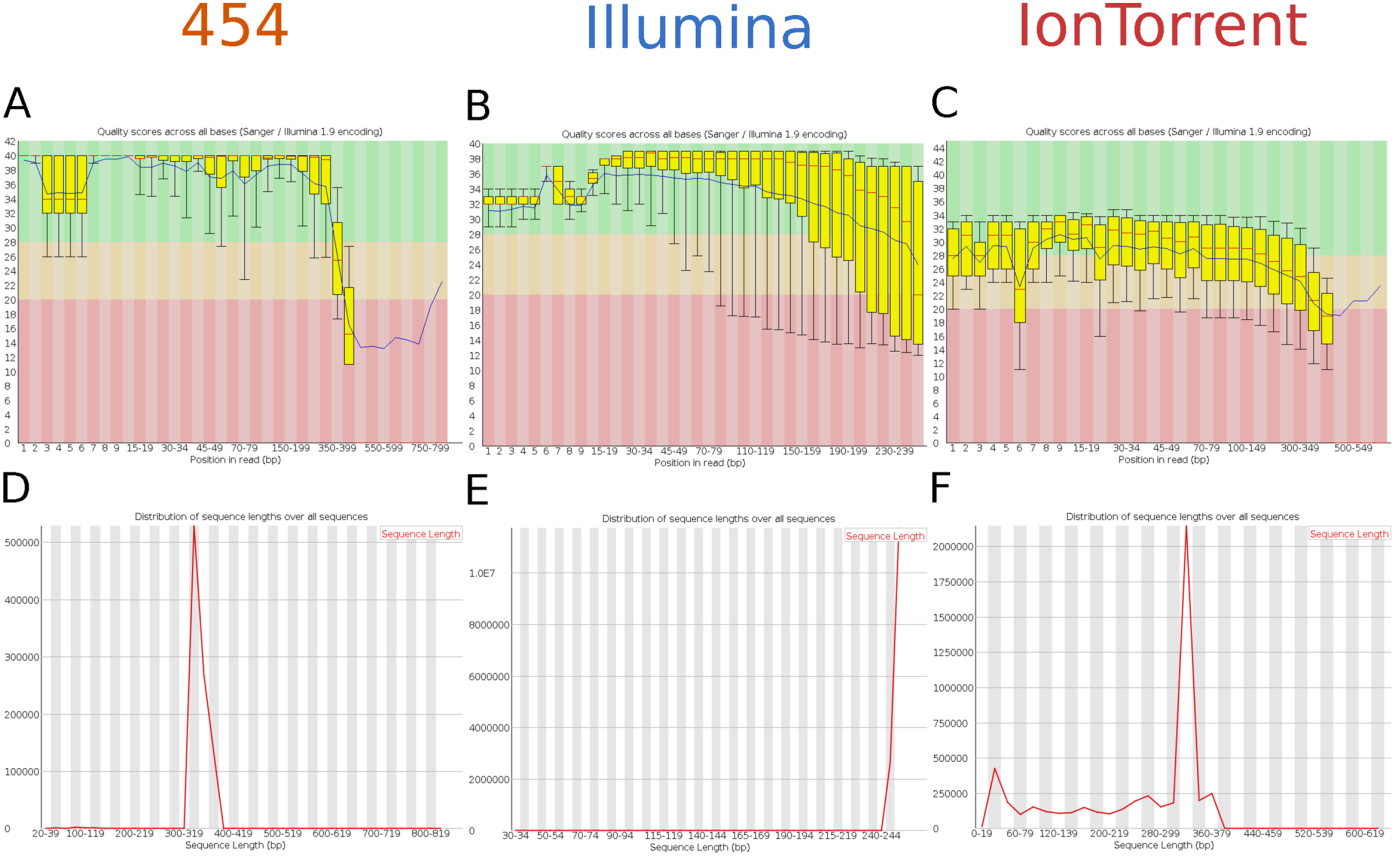
Platform comparison—base quality & read length quality assessment. Per base quality scores and read length of all three platforms are shown for 454 (A, D), Illumina (B, E), and IonTorrent (C, F) using FASTQC algorithm.

To analyse the quality of the reads among single read positions, all base positions were plotted against the quality score by using FastQC. The results in Fig 3 demonstrate that 454 has an overall high Phred score within base position 7–300 bp of the sequenced amplicon libraries, with a strong decrease after base position 350 (Fig 3A). According to a short homopolymeric G base stretch of 4 bp in the 5’ linker sequence we observed a dropdown of the Phred score at base position 4–7 for 454 reads. In contrast to the 454 platform, Illumina MiSeq data output resulted in a stable Phred score (~ Q38) among the entire read length (Fig 3B). The read length distribution clearly demonstrated that 454 reads are in the expected size range (300–400 bp) of the sequenced amplicons (Fig 3D), whereas Illumina reads were limited by the sequencing chemistry of 250 bp (Fig 3E). In PGM minor fractions of shorter reads were detected beside the major read length of 325 bp (Fig 3F).

Concerning the results of Phred score distribution among all bases the PGM data showed the lowest overall Phred values for the sequencing run (Fig 3C), with a high number of short reads, compared to the other platforms (Fig 3D–3F). In contrast both GS FLX and MiSeq data yielded high Phred scores (Fig 3A and 3B)—with only minor reduction of Phred base quality during the end of the runs.

### Platform-specific sequencing errors

To investigate platform-specific error types and error rates, reads of each platform were aligned against the CREBBP reference sequences. The major error types occurring during the entire deep sequencing process, originated from three different sources (i) PCR-derived errors; (ii) HTS platform-derived errors, and (iii) experiment/run-specific errors that lead predominantly to point mutations and/or Indels. Since the CREBBP mutation profile of the analysed samples was unknown, we performed conventional Sanger sequencing, demonstrating that no dominant mutations were detectable in respective samples that could influence error rate estimation (data not shown).

After alignment of all reads against the reference sequences error rates for substitutions and Indels for each platform were determined. Substitution errors ranged from 0.07% (0.24 subs/read) for 454 generated sequences, up to 1.53% (3.67 subs/read) in sequences derived from the Illumina MiSeq run (Table 2). In contrast Indel specific errors were mainly present in IonTorrent PGM reads with a frequency of 0.60% (1.63 Indels/read), while the substitution specific errors are 0.13% (0.37 subs/read). Analysis of the total read number that could be aligned to a reference sequence resulted in 99.75% for 454 reads, 98.1% for Illumina reads and 90.65% for PGM deriving reads.

**Table 2:** Platform comparison—error rates.

Platform	aligned bases	aligned reads	reads %	subst	subst read	subst %	Indels	Indels read	Indels %
**454 GS FLX**	270,606,795	801,259	99.75	191,942	0.24	**0.07**	460,650	0.59	**0.18**
**IonTorrent PGM (318)**	1,099,847,452	4,386,444	90.65	1,608,351	0.37	**0.13**	6,474,165	1.63	**0.60**
**Illumina MiSeq**	3,200,215,421	12,807,147	98.1	50,070,350	3.67	**1.53**	1,197,803	0.09	**0.04**

The MiSeq data showed the highest substitution error rate and PGM data revealed the highest Indel error rate, with the largest portion of data that could not be aligned against the reference data set.

According to differences in the local base composition (e.g. homopolymers; GGC sequences, inverted repeats) of the reference sequences, we analysed substitution and Indel errors individually for each amplicon and sequencing run.

**Fig 4:**
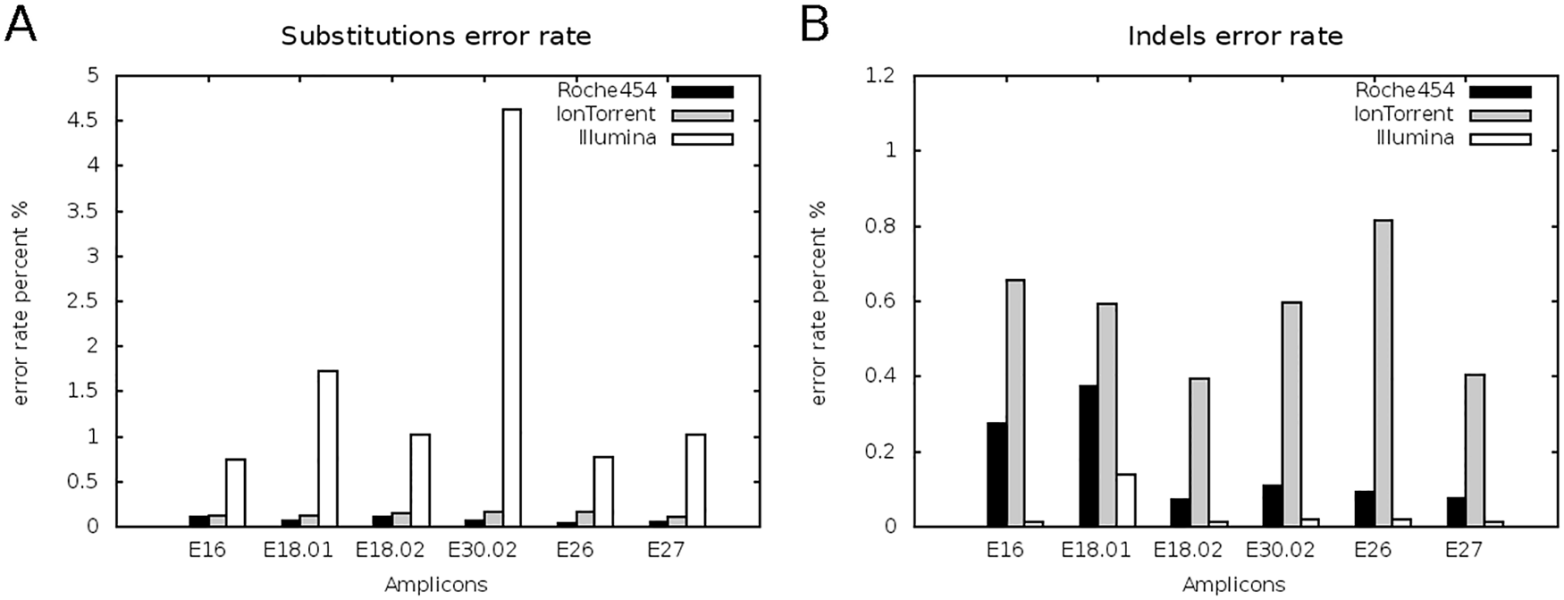
Platform comparison—amplicon-specific error rates. HTS platform-specific substitution (A) and Indel (B) error rates for amplicon libraries sequenced by 454 (black), Illumina (white), and IonTorrent (light grey) platform are displayed for single amplicons covering corresponding exons of the CREBBP gene.

The data in Fig 4A show that there was an overall high substitution error (up to 4.6% in Exon 30) of reads deriving from the MiSeq run, independently of the reference analysed. Both, GS FLX and PGM data resulted in a very low substitution error rate (< 0.5%) for all amplicons. High frequency Indel (0.8% in Exon 26, Fig 4B) were only found in run of PGM. Indel errors of GS FLX range 0.1–0.4%; very low Indel errors (<0.1%) were detectable in MiSeq data.

The highsubstitution error rate of the MiSeq was dependent on the sequence context of the respective amplicon (Fig 4A). Additionally, Indel errors seem to occur independently of the sequence motif in PGM data, where high Indel errors are detectable for each amplicon (Fig 4B).

To gain additional information on substitution errors resulting from the sequencing runs, especially from Illumina MiSeq data, we performed detailed analysis using a substitution matrix. Homopolymeric and GC-rich regions were excluded from analysis to define a representative error rate for each base substitution.

Base-specific substitution errors for GS FLX and PGM ranged from 0.01 to 0.03% (Table 3), with transitions being more frequent than transversions (transversions were not detectable in GS FLX data; 0–0.01% in PGM data). The specific error rate followed the order A/T>G>C in PGM, whereas in GS FLX data total errors occured base-independently. MiSeq sequences mainly showed transversion errors with a frequency of 0.05–0.1%; compared to transition errors 0.02–0.05%. Bases A/T were more prone to substitutions with a total error rate of 0.16–0.17%, compared to G/C bases with a lower total error rate of 0.09–0.1%.

**Table 3:** Platform comparison—Base-specific error rates.

Correct base	Read as:				
**GS FLX run**	**A**	**C**	**G**	**T**	**Total**
**A**		0.00%	***0*.*02%***	0.00%	0.02%
**C**	0.00%		0.00%	***0*.*01%***	0.01%
**G**	***0*.*01%***	0.00%		0.00%	0.01%
**T**	0.00%	***0*.*01%***	0.00%		0.01%
**MiSeq run**	**A**	**C**	**G**	**T**	**Total**
**A**		**0.09%**	*0*.*05%*	0.02%	0.16%
**C**	**0.06%**		0.01%	*0*.*02%*	0.09%
**G**	*0*.*03%*	0.02%		**0.05%**	0.10%
**T**	0.03%	*0*.*04%*	**0.10%**		0.17%
**PGM run**	**A**	**C**	**G**	**T**	**Total**
**A**		0.00%	***0*.*02%***	0.01%	0.03%
**C**	0.00%		0.00%	***0*.*01%***	0.01%
**G**	***0*.*02%***	0.00%		0.00%	0.02%
**T**	0.00%	***0*.*02%***	0.01%		0.03%

## Discussion

In this study, we evaluated the ability of three major deep sequencing platforms 454 GS FLX (Roche), MiSeq (Illumina), and IonTorrent PGM (Life Technologies) on sequencing well characterized single reference gene amplicons that were generated in a universal two-step PCR approach, thereby reducing PCR bias to obtain comparable results for platform-related error rates.

Recent studies compared performance of deep sequencing platforms by analysing data of viral or bacterial genomes using genomic library preparation methods [8,20]. However, only rare information exists on platform-comparisons of amplicon-based library preparations, focusing on distinct genes. Here we demonstrate a stable two-step PCR approach to generate amplicon-libraries for the three major HTS platforms currently available. According to several sources of errors before and during the deep sequencing procedure, like DNA extraction, PCR amplification of target regions, library preparation and sequencing, we aimed to standardize these steps prior the sequencing reactions. We started with a unique first-round amplification and prepared the gene-specific amplicons with specific linker sequences using hybrid primers to provide a binding site for platform-specific PCR primers. A major advantage of our proposed method is to avoid several amplicon-library preparation methods (e.g. adapter ligation) that are specific for each sequencing platform, thereby requiring a large amount of genomic DNA, different primer sets and various library preparation protocols. In case of multiplexed/barcoded samples a large number of primers is necessary for each sample in complex sequencing assays with multiple reactions; whereas in the two-step PCR approach only the second-round PCR primers have to be barcoded independent of the number of reactions within the first-round PCR. Nevertheless, PCR amplification prior sequencing or during library preparation is susceptible to amplification bias/errors and thus could alter the right interpretation of sequencing results of the different platforms. An alternative way to overcome PCR bias is to avoid or limit amplification steps in sample preparation methods by using new single molecule sequencing technologies, like Oxford Nanopore GridION or Pacific Bioscience platform [21,22]. In our study we used a consistent experimental design and reduced the PCR bias by using a proofreading enzyme for amplification with a low overall error rate (0.23%) tested before in a 454 sequencing assay of TP53 plasmids [23].

Sequencing data revealed from all three platforms were analysed according to read number, predicted quality and length of reads. The results were similar to previous studies, demonstrating that MiSeq produces the highest throughput (3.7 Gb); PGM shows the shortest reads (248 bp), and the GS FLX has the lowest throughput (0.3 Gb) with the longest reads (337 bp) [8,24]. By analysing Phred quality distribution among the entire read lengths, we observed a dropdown of the Phred scores at base position 4–7 in 454 reads, according to a short homopolymeric G base stretch of 4 bp in the 5’ linker sequence (Fig 3). This result indicates that 454 specific errors are induced even by short stretches of identical bases [11]. In GS FLX and MiSeq reads we also detected a minor reduction of Phred quality scores during the end of the runs, according to the sequencing chemistry used.

After alignment of the filtered reads we determined platform-specific error rates specific for our amplicon sequencing approach. Several studies focused on platform-specific errors for whole-genome sequencing data. For example for GS FLX error rates of 0.28% for Indels and 0.12% for substitutions (max. 1.07%) were reported [25]. In GS20 sequencer produced data error rates reached <0.1–0.49%, and Margulies described error rates in range 0.6–4% [1,11]. Lower error rates derived from Roche test-fragments that did not undergo library preparation or PCR amplification steps [26]. Also for PGM data error rates around 1.5% for Indels were reported, which was be confirmed by another study revealing errors of ~1.8% [8,25]. In both platforms Indel errors derived from homopolymers with a maximum local error rate in GS FLX up to 50% [26,27]. Our data show low Indel and substitution error rates (subs 0.07%; Indel 0.18%), using GS FLX- comparable to the results of McElroy et al., whereas in PGM derived data Indel errors (0.6%) were 2.5-3-fold lower compared to other studies [25]. analysis potential explanation for this discrepance is that in our study defined amplicon sequences with a small diversity were analysed whereas other published data focus on whole genome sequencing [8,9]. We observed higher Indel error rates for the amplicons E16 and E18.01 that correlate to 7 A and 13 T base stretches, respectively (Fig 4B).

MiSeq-specific errors were described as substitutions with a frequency of around 0.1%, and Indels reaching an insignificant rate < 0.001% [8]. However, depending on the sequence context (e.g. inverted repeats, GGC sequences) also higher Illumina error rates of 0.31 to 1.66% have been described [28] with a maximum of up to 6% in GC-rich motifs [25,29]. Here we report a higher mean substitution rate of 1.53% with amplicon-specific differences; (Fig 4A) in amplicon E30.02 error rate reached ~4.5%

To identify the source of substitution errors, we generated a substitution matrix for all three runs. Shao *et al*. describe that transitions were 5–10 fold more frequent than transversions in 454 derived data, whereas transitions ranged from 0.04–0.1% and transversions from 0–0.03%. The specific error rate followed the order A≥T>G>C [20]. We found 454 base-specific error rates of 0.00–0.02%, with only transitions being detectable. This result clearly demonstrates that substitutions are only a minor source of error for the 454 platform. Analysing the PGM reads we found base-specific errors ranging from 0.00–0.03% comparable to the 454 results; also Indel errors are the dominant source of errors for the reads. Substitution error rate of PGM reads was reported between 0.04–0.1% depending on the kit used for sequencing reaction; also showing that A and T flows are more error-prone cycles. This is in agreement with our data with a total error of 0.03% each for A and T substitutions [30]. The high substitution error rates in Illumina-derived data result mainly from A <-> C (0.15%) and T <-> G (0.15%) substitutions.

## Conclusions

Summarizing our data, we demonstrate that each of the three sequencing platforms is characterized by its specific error rate and type of errors. The two step-amplification approach described here shows that amplicons deriving from a single source of DNA can be sequenced in parallel by different platforms. Specific errors occurring due to the amplicon preparation process can be minimized by combining the different results. In our study Illumina had the highest error rate for substitution errors, but the lowest number of Indel errors in single amplicons. Thus could be counterbalanced by data of the 454 run, which show the lowest number of substitutions but the highest Indel error rate. Here we propose a method avoiding expensive and time-consuming platform-specific library preparation kits (e. g. adapter ligation protocols), and reducing specific amplification errors during different library preparation procedures. Furthermore, this method can be easily adapted to emerging amplicon-based sequencing technologies in future.
